# Evaluation of the Necessity of a Cleanup Step After Microwave-Assisted Extraction (MAE) of Aflatoxins in Pea Flour

**DOI:** 10.3390/molecules31122035

**Published:** 2026-06-10

**Authors:** Laura Barp, Elisa Denittis, Chiara Conchione, Sabrina Moret

**Affiliations:** Department of Agri-Food, Environmental and Animal Science, University of Udine, via Sondrio 2/a, 33100 Udine, Italychiara.conchione@uniud.it (C.C.); sabrina.moret@uniud.it (S.M.)

**Keywords:** aflatoxins, *Pisum sativum*, microwave-assisted extraction (MAE), solid-phase extraction (SPE), HPLC-FLD

## Abstract

Aflatoxins (AFs) are among the most hazardous mycotoxins in food, yet their occurrence in legume-based products remains poorly investigated, and official standardized analytical methods validated by international regulatory bodies are still limited for these specific matrices. This study evaluates the effectiveness of microwave-assisted extraction (MAE) coupled with HPLC-FLD for the determination of AFB1, AFB2, AFG1, and AFG2 in pea flour (*Pisum sativum*), with a particular focus on assessing the necessity of a solid-phase extraction (SPE) cleanup step. Two analytical workflows were compared: direct MAE and MAE followed by SPE purification. The optimized MAE conditions (60 °C, 5 min, acidified acetonitrile) provided good repeatability (RSD < 8.7%) and acceptable recoveries (70–73% at 10 µg/kg), while the inclusion of SPE improved recoveries up to 95–103% but did not yield a noteworthy reduction in matrix effects for AFB1 and AFG1. Matrix-induced signal suppression (up to −24.6%) was observed in both approaches, highlighting the necessity of matrix-matched calibration. Limits of quantification ranged from 0.09 to 0.25 µg/kg (MAE) and from 0.12 to 0.43 µg/kg (MAE + SPE), with no statistically significant differences between the two protocols. Application to commercial pea flours (*n* = 7) revealed no quantifiable contamination, with only one sample showing AFB1 below the LOQ. Overall, the results demonstrate that under the tested conditions, the direct MAE approach, combined with trifluoroacetic acid derivatization and HPLC-FLD analysis, provides a reliable and efficient alternative to conventional cleanup-based methods for routine AF analysis in pea flour. The application to a small batch of commercial samples confirms the method’s fitness-for-purpose as a preliminary screening tool.

## 1. Introduction

Mycotoxins are low-molecular-weight secondary metabolites produced by various fungal species and represent a major concern for food safety due to their high toxicity and widespread occurrence in agricultural commodities [1,2]. Among these, aflatoxins (AFs), primarily produced by *Aspergillus flavus* and *Aspergillus parasiticus*, constitute the most critical group. AFs, particularly B1, B2, G1, and G2, pose serious risks to human and animal health, ranging from acute toxicity to immunosuppression and potent carcinogenicity [3,4]. Notably, aflatoxin B1 (AFB1) is categorized as a Group 1 carcinogen by the International Agency for Research on Cancer (IARC, 1993) [5]. Consequently, their presence in the food supply chain threatens public health and causes significant economic losses globally [6].

While AFs are traditionally associated with cereals, oilseeds, and nuts, their occurrence in pulses is receiving increasing attention. Grains and grain-based products remain the primary source of chronic dietary exposure to AFB1 [3], but recent shifts in consumer habits, particularly the rise in plant-based diets, have highlighted new risk profiles [7,8,9]. Although Regulation (EU) 2023/915 [10] establishes strict maximum levels for AFs in various food groups, legumes are not yet specifically included [2].

The global demand for pea protein (*Pisum sativum*) is rapidly expanding, with the market projected to reach $47.16 billion by 2031 [11]. Although fungal contamination in beans and peas poses a documented risk to consumer health [12,13], occurrence data for these matrices remain scarce [2]. Legumes present major analytical challenges: the high protein content (~20–25%) and complex starch/fiber structure promote strong toxin-matrix interactions, hindering mass transfer during conventional extraction [14]. Consequently, official protocols such as EN ISO 16050:2011 [15] or Association of Official Analytical Chemists (AOAC) methods, designed for cereals, nuts, and spice-based pastes, are not suitable for pea flour, as standard solvent mixtures can induce starch swelling and protein precipitation. This phenomenon can trap AFs within the matrix, resulting in poor recoveries and severe analytical interferences.

While conventional workflows rely on moderately polar mixtures, such as methanol/water or acetonitrile/water [16,17], followed by laborious solid-phase extraction (SPE) or immunoaffinity cleanup, modern analytical chemistry prioritizes sustainable, high-throughput approaches [14]. Microwave-assisted extraction (MAE) addresses these needs by offering rapid extraction, automation, and lower solvent consumption [18]. Although MAE has been successfully applied to grains [19], apples [20], and corn flour [21], its performance on protein-rich legumes, such as pea flour, remains virtually unexplored, particularly regarding the objective necessity of a post-extraction cleanup step.

Therefore, this study aims to investigate an MAE-based method for the simultaneous determination of the four main aflatoxins in pea flour, focusing on whether a post-extraction SPE cleanup step is strictly required or whether a direct workflow can deliver compliant and reliable analytical performance.

## 2. Results

### 2.1. Optimization of the Extraction Procedure

#### 2.1.1. Microwave-Assisted Extraction (MAE)

The efficiency of the MAE process was evaluated by comparing two protocols adapted from the literature. Set 1, based on the method described by Chen and Zhang [19] for cereal grains (80 °C, 15 min, pure acetonitrile), resulted in unsatisfactory performance for AFB1 and AFG1, with apparent recoveries reaching approximately 150% and poor precision (RSD > 30%). Conversely, Set 2, adapted from the multi-residue approach of Kalogiouri et al. [20] (60 °C, 5 min), involving the addition of 20 mL of acetonitrile acidified with acetic acid (1%, *v*/*v*), demonstrated significantly higher stability, yielding consistent recoveries between 115% and 124% and superior repeatability (RSD < 5.1%).

#### 2.1.2. Cleanup

Initial SPE trials using a 4 mL loading volume resulted in recoveries below 12%. Following optimization, reducing the loading volume to 600 µL (400 µL sample + 200 µL dichloromethane washes) significantly enhanced efficiency, yielding recovery values between 105% and 110% (*n* = 3, RSD < 4.5%).

#### 2.1.3. Acid Hydrolysis

Direct addition of 15 µL trifluoroacetic acid (TFA) to the dry residue effectively enhanced the fluorescence of AFB1 and AFG1. This acid-catalyzed hydration selectively adds a hydroxyl group across the terminal furan double bond of AFB1 and AFG, converting them into the more polar hemiacetals. This increased polarity weakens their hydrophobic interactions with the C18 stationary phase, shifting the elution order from G2-G1-B2-B1 (untreated) to G1-B1-G2-B2 (TFA-treated), as AFB2 and AFG2 lack the reactive double bond and remain unchanged.

### 2.2. Method Performance

Calibration parameters for AFs in solvent and matrix-matched solutions (MAE and MAE + SPE) are summarized in Table 1. All curves exhibited excellent linearity with coefficients of determination (R^2^) ≥ 0.9985. A lack-of-fit test confirmed the adequacy of the linear model (*p* > 0.05). Significant signal suppression (ME < −20%) was observed for AFG1 and AFB1 in both protocols. Specifically, the SPE cleanup did not significantly mitigate suppression for AFG1 and AFB1, with matrix effect (ME) values shifting from −24.6% and −23.3% (MAE) to −28.9% and −21.4% (MAE + SPE), respectively (Student’s *t*-test, *p* > 0.05). Conversely, for AFG2 and AFB2, the SPE step reduced the ME, already well within the acceptable threshold of ±20% typically defined by international guidelines [22], from approximately −11% to negligible values (+0.4% and −2.1%, respectively).

The precision of both protocols was evaluated at three fortification levels (0.4, 2.0, and 10.0 µg/kg). As summarized in Table 2, all intra-day (RSD_r_) and inter-day (RSD_R_) precision values were well below the maximum thresholds established by Commission Implementing Regulation (EU) 2023/2782 (RSD_r_ ≤ 20% and RSD_R_ ≤ 30% for mycotoxin concentrations ≤ 10 µg/kg) [23].

For the MAE protocol, intra-day RSD% ranged from 0.2% to 5.1%, while inter-day precision remained below 8.7% for all analytes. The MAE + SPE protocol exhibited slightly lower inter-day variability, with RSD% values in the 0.4–6.6% range.

The extraction efficiency was evaluated at two fortification levels (2.0 and 10.0 µg/kg). For the MAE protocol, recoveries at the 2.0 µg/kg level ranged from 65.7 ± 12.6% (AFB2) to 89.7 ± 14.4% (AFG1). At the higher fortification level (10.0 µg/kg), the recovery values became more uniform, consistently falling within the 70.2 ± 8.0% to 73.0 ± 0.8% range (Table 2).

The inclusion of the SPE cleanup (MAE + SPE) led to an overall increase in recovery rates. At 2.0 µg/kg, recoveries ranged from 83.1 ± 4.1% (AFG2) to 98.8 ± 1.9% (AFB1), while at 10.0 µg/kg, the values were nearly quantitative, ranging from 95.2 ± 4.6% (AFG2) to 103 ± 11% (AFG1) (Table 2). All results, regardless of the protocol used, were found to be compliant with the performance criteria established by Commission Regulation (EU) 2023/2782 [23], which typically considers recoveries between 70% and 120% as acceptable for this concentration range.

The Limits of Detection (LOD) and Quantification (LOQ) for both procedures are summarized in Table 3. The comparison revealed a nominal increase in LOQ values for all four AFs when the SPE step was included, deviating from conventional analytical expectations. For instance, the LOQ for AFG1 rose from 0.25 µg/kg in the MAE protocol to 0.43 µg/kg in the MAE + SPE protocol. However, the application of the critical difference test revealed that these increases were not statistically significant (*p* > 0.05). For all four analytes, the absolute difference between the two methods (|Δ_LOQ_|) remained strictly below the calculated combined expanded uncertainty (U_comb_), demonstrating that the observed discrepancies fall within the inherent measurement uncertainty of the analytical system. Despite the nominal increase in the MAE + SPE protocol, the LOQs for all AFs in both methods remained well below the maximum levels established by Commission Regulation (EU) 2023/915 [23] (e.g., ≤1.0 µg/kg for all foods other than baby foods).

### 2.3. Occurrence of AFs in Commercial Pea Flours

To evaluate the practical applicability of the optimized MAE-HPLC-FLD method, seven commercial pea flour samples were analyzed. The results showed that aflatoxin contamination in the surveyed products was extremely low. Notably, only one sample (FP5) exhibited a trace detection of AFB1 below the LOQ, with an estimated semi-quantitative concentration of 0.08 µg/kg and a signal-to-noise ratio (S/N) of 4.5, confirming a true positive presence below the formal quantification threshold but above the LOD (0.05 µg/kg). In all other samples (FP1–FP4, FP6–FP7), aflatoxins were not detected or were below the LOD.

## 3. Discussion

### 3.1. Considerations on the Extraction Procedure

The primary challenge in this study was the extraction parameters. The overestimation observed in Set 1 (80 °C) suggests that high temperatures and pure acetonitrile co-extract polar interferences from the matrix, which likely cause signal enhancement in the FLD. The transition to Set 2 (60 °C with 1% acetic acid) was decisive. The acid seems to act as a disruptor of the protein-aflatoxin complexes, allowing for efficient release at lower temperatures, thus minimizing the co-extraction of interfering macromolecules.

The initial SPE failure (<12% recovery) was likely due to a breakthrough effect caused by loading the sample in 4 mL of dichloromethane. According to normal-phase SPE principles, analytes should be loaded in very weak solvents (e.g., hexane) to maximize retention via hydrogen bonding between the AFs’ carbonyl/furan rings and active surface silanols. However, the poor solubility of AFs in alkanes restricts the choice. Although dichloromethane ensures solubility, it possesses a non-negligible solvent strength on silica and acts as a competitive eluent if used in high amounts (4 mL), displacing the analytes from the active sites. Minimizing the loading volume to 600 µL effectively resolved this competition, preserving quantitative analyte retention via dipole–dipole and H-bonding interactions while preventing premature elution.

The superiority of the direct TFA derivatization [24] over the heated hexane-mediated protocol [19] can be rationalized by the enhanced reaction kinetics occurring at the interface of the dry extract residue. In the absence of a bulk non-polar solvent like hexane, in which AFs exhibit poor solubility, the TFA acts both as a catalyst and a concentrated medium, ensuring the quantitative conversion of AFB1 and AFG1 into their highly fluorescent hemiacetal derivatives (AFB2a and AFG2a).

This chemical transformation is further confirmed by the significant shift in chromatographic selectivity. The introduction of a hydroxyl group during the acid-catalyzed hydration increases the analytes’ polarity, weakening their hydrophobic interactions with the C18 stationary phase [25].

Beyond derivatization, the addition of concentrated TFA (a strong organic acid) to the raw MAE extract induces a concomitant precipitation of matrix interferents. The acid treatment promotes the pheophytinization of chlorophylls (the displacement of the central Mg^2+^ ion), which alters pigment solubility, while simultaneously causing the denaturation and subsequent macroscopic precipitation of co-extracted residual pea proteins [26]. Once these suspended aggregates are removed via 0.22 µm PTFE filtration, the resulting extract is remarkably clean. This mechanism is quantitatively corroborated by the matrix effect (ME%) data reported in Table 1; for the non-reactive analytes (AFB2 and AFG2), the direct MAE approach yields matrix mitigation values that are statistically equivalent to those obtained with the multi-step SPE cleanup, demonstrating that this acid-induced precipitation sufficiently minimizes background interferences for routine analysis.

### 3.2. Method Performance and Protocols Comparison

#### 3.2.1. Matrix Effect

The experimental data (Table 1) highlight a clear divergence between the chromatographic behavior of AFs in pure solvent and in *Pisum sativum* extracts. In the MAE-only protocol, the slopes of the calibration curves for all four analytes were significantly lower than those obtained in solvent, with signal suppression reaching −24.6% for AFG1 and −23.3% for AFB1. Even for the less affected AFG2 and AFB2, the suppression remained around −11%, which, although within some legal tolerances, still represents a non-negligible analytical bias.

The comparison of matrix effects between the MAE and MAE + SPE protocols (Table 1) reveals a selective behavior of the *Pisum sativum* matrix. While the SPE cleanup successfully neutralized the matrix effect for AFG2 and AFB2 (shifting from ~−11% to negligible values), it failed to mitigate the significant signal suppression for AFG1 and AFB1 (ME < −21%).

**Figure 1:**
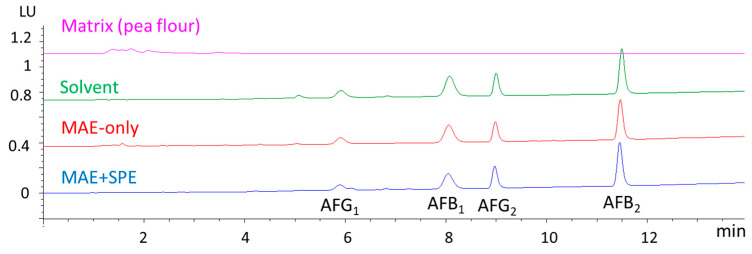
Stacked HPLC-FLD chromatograms of pea flour analysis: (a) unfortified matrix blank (pink trace); (b) standard solution in solvent (green trace, 2 µg/kg for each AF); (c) fortified matrix extract without cleanup (red trace, 2 µg/kg for each AF, “MAE-only”); and (d) fortified matrix extract with SPE cleanup (blue trace, 2 µg/kg for each AF, “MAE + SPE”).

A visual comparison of the chromatographic responses is reported in Figure 1 and Figure S1.

This phenomenon can be explained by the chemical nature of the co-extracted interferents. The protein-rich nature of pea flour likely leads to the co-extraction of small, polar nitrogenous compounds or peptides that survive the silica-based SPE. Since AFG1 and AFB1 (as their TFA-derivatives) elute earlier in the chromatogram compared to their saturated counterparts (G2 and B2), they are more susceptible to overlap with these early-eluting polar matrix components. The protein-rich nature of pea flour leads to the co-extraction of small, polar nitrogenous compounds or peptides that survive silica-based extraction. Because unmodified silica cartridges operate via a normal-phase mechanism, retention is driven by hydrogen bonding with surface silanol groups. These co-extracted polar matrix components possess a heteroatom density similar to the lactone and carbonyl structures of AFs, leading to identical retention and co-elution during cleanup. Consequently, the adoption of matrix-matched calibration becomes a statistical necessity to ensure accuracy. In fact, using standard solvent-based calibration curves would result in a severe underestimation of AFB1 and AFG1 concentrations (exceeding 23%), introducing an unacceptable systematic error that invalidates legal compliance evaluations. To circumvent this specific co-elution, alternative cleanup strategies such as polymer-based Hydrophilic-Lipophilic Balanced (HLB) cartridges or highly specific Immunoaffinity Columns (IAC) could be employed to effectively differentiate these matrix interferences from the target mycotoxins based on hydrophobic balances or biological affinity.

Nevertheless, matrix-induced signal suppression in fluorometric detection may arise not only from co-eluting compounds but also from fluorescence quenching or inner filter effects.

#### 3.2.2. Precision, Recovery, and Sensitivity

Both protocols demonstrated excellent ruggedness, with RSD% values (Table 2) consistently lower than the maximum limits allowed by Reg. (EU) 2023/2782 [23]. This confirms that the MAE process provides a highly repeatable extraction across different fortification levels.

Regarding recoveries, the inclusion of SPE (MAE + SPE) led to nearly quantitative results (~95–103%). However, the direct MAE protocol, despite lower recovery values (~70–73% at 10 µg/kg), remains fully compliant with the legal threshold (70–120%) [23]. The marginal gain in recovery provided by SPE does not justify the additional processing time and the consumption of chlorinated solvents, especially for routine screening where throughput is a priority.

A striking result of this study is the nominal increase in LOQ values upon incorporating the SPE step (e.g., from 0.25 to 0.43 µg/kg for G1). Generally, sample cleanup is expected to lower the LOQ by lowering baseline noise. However, because the optimized MAE protocol yielded an inherently clean extract with minimal background interference, the chemical noise reduction provided by the SPE was negligible. According to error propagation, this heightened experimental variability outweighs the baseline benefits, directly leading to elevated LOQ values.

The application of the critical difference test (|ΔLOQ| < U_comb_) proves that the two methods are metrologically equivalent. The fact that the LOQs for the MAE-only method are equal to or lower than those of the MAE + SPE method, and all are well below the strict EU limits for cereals and legumes (≤1.0 µg/kg), is a powerful argument for avoiding the cleanup approach. The native selectivity of the HPLC-FLD system, combined with the chemical cleanup provided by TFA, seems sufficient to reach the required sensitivity in pea flour.

### 3.3. Aflatoxin Contamination in Commercial Pea Flours

The low incidence of AFs observed in this survey is consistent with the limited literature available on *Pisum sativum*. While legumes like the common bean (*Phaseolus vulgaris*) have been extensively studied [12], data on pea flour remains scarce. Recent reports on whole pulses from other geographic regions, such as Bhutan, found higher mean values of AFB1 (44.83 µg/kg) in split peas and lentils [27]. This discrepancy could be attributed to differences in climatic conditions, storage practices, and the industrial processing involved in flour production, which may mitigate or dilute fungal contamination.

Although the small sample size evaluated in this study (*n* = 7), it is important to emphasize that the primary scope of this work was the development and validation of the analytical method, rather than a comprehensive monitoring survey. This small sampling pool also reflects the current market status of pea flour, which remains a niche foodstuff predominantly restricted to specialized dietary sectors. Nevertheless, this restricted sample size represents an acknowledged limitation for assessing widespread contamination trends. As the market demand for plant-based protein alternatives continues to expand rapidly, a larger-scale, multi-regional market survey will be essential in future investigations to comprehensively map mycotoxin prevalence along the legume flour supply chain and support regulatory monitoring under Regulation (EU) 2023/915 [10].

## 4. Materials and Methods

### 4.1. Reagents and Standards

HPLC-grade acetone, acetic acid, formic acid, chloroform, dichloromethane, and *n*-hexane were purchased from Merk (Darmstadt, Germany). Acetonitrile and methanol (HPLC-grade) were supplied by BDH Laboratory Supplies (Poole, UK). Ethyl ether was purchased from Carlo Erba Reagents (Milan, Italy), and TFA was sourced from Biosolve B.V. (Valkenswaard, the Netherlands). High-purity water (18.2 MΩ·cm) was produced from a Milli-Q Advantage A10 water purification system (Merck, Darmstadt, Germany).

Individual AF standards (AFB1, AFB2, AFG1, AFG2) at 100 µg/mL in acetonitrile were purchased from Affinisep (Le Houlme, France). A certified multi-component standard solution (AFB1, AFB2, AFG1, AFG2, 10 µg/mL each in acetonitrile) was supplied by Restek (Bellefonte, PA, USA). Stock solutions were stored at −20 °C in amber glass vials to prevent photodegradation. Working solutions were prepared via appropriate dilution in acetonitrile and kept under the same storage conditions.

### 4.2. Samples

Eight commercial pea flours were purchased from retail markets in Italy and Slovenia. The samples were coded, and their place of production, market placement, and nutritional composition, as declared by manufacturers, are summarized in Table 4. The samples exhibited significant variability in protein, fiber, fat, and carbohydrate content, reflecting potential differences in processing methods, geographical origin, and pea varieties. The organic pea flour (FP1) was selected for method optimization and validation due to its representative nutritional profile and the absence of detectable endogenous AFs, ensuring a reliable blank matrix for recovery and sensitivity studies (Figure S2). All samples were stored at room temperature in a dry, dark place and homogenized before analysis.

### 4.3. Aflatoxin Determination

#### 4.3.1. Microwave-Assisted Extraction (MAE) and SPE Purification

The extraction procedure was adapted from Kalogiouri et al. [20] and optimized for the legume matrix. Briefly, 5 g of pea flour was weighed into an HP-500 TFM vessel (CEM Corporation, Matthews, NC, USA), and 20 mL of acetonitrile acidified with acetic acid (1%, *v*/*v*) was added. Extraction was performed using a MARS6 microwave system (CEM Corporation, Matthews, NC, USA) at 60 °C and 350 psi for 5 min, with a 5 min ramp time and continuous magnetic stirring. Following the extraction cycle, vessels were cooled to 30 °C before opening.

To critically evaluate the impact of matrix interference and assess the actual necessity of a purification step, the raw extract was processed following two distinct analytical pathways:

- Direct MAE protocol: a 4 mL aliquot of the raw extract (equivalent to 1 g of sample) was evaporated to dryness under vacuum. The residue was directly subjected to pre-column derivatization (Section 4.3.2) and subsequent HPLC-FLD analysis.
- MAE + SPE protocol: a second 4 mL aliquot of the raw extract was subjected to SPE purification based on the protocol by Chen & Zhang [19]. The residue was reconstituted in 400 µL of dichloromethane and loaded onto a Sep-Pak silica cartridge (690 mg, Waters, Milford, MA, USA), previously conditioned with 5 mL of dichloromethane. To ensure quantitative transfer, the sample vial was rinsed twice with 100 µL of dichloromethane. The cartridge was sequentially washed with 5 mL each of *n*-hexane, ethyl ether, and dichloromethane to remove matrix interferences. AFs were finally eluted with 8 mL of the chloroform/acetone mixture (4:1, *v*/*v*). The eluate was evaporated to dryness, followed by derivatization (Section 4.3.2) and HPLC-FLD analysis.

#### 4.3.2. Pre-Column Derivatization

To enhance the fluorescence of AFB1 and AFG1, pre-column acid-catalyzed derivatization was performed according to Takahashi [24]. The dry residue was treated with 15 µL of TFA and vortexed for 30 s. The mixture was then reconstituted with water/acetonitrile (90:10, *v*/*v*) to reach the final volume of 1 mL. The resulting solution was filtered through a 0.22 µm PTFE syringe filter (Lab Logistics Group GmbH, Meckenheim, Germany) directly into an amber HPLC vial for subsequent chromatographic analysis.

#### 4.3.3. HPLC-FLD Conditions and Quantitative Analysis

Chromatographic analysis was performed on an Agilent 1260 Infinity HPLC system equipped with an FLD (Agilent Technologies, Santa Clara, CA, USA). Separation was achieved using a Poroshell 120 SB-C18 column (4.6 mm × 100 mm, 2.7 µm; Agilent Technologies) maintained at 40 °C. The mobile phase consisted of 0.2% aqueous formic acid and methanol, delivered at a constant flow rate of 0.8 mL/min. The gradient was initiated at 30% methanol (held 1 min), increased to 50% over 12 min, and then ramped to 90% (3 min) to ensure effective column washing. Finally, the column was reconditioned to the initial conditions for 3 min before the next injection. The injection volume was 5 µL. The FLD was operated at λ_ex_ = 360 nm and λ_em_ = 440 nm.

Data acquisition and processing were performed using OpenLAB CDS ChemStation Edition software (version 1.4.209, Agilent Technologies). Peak identification was based on the retention times of individual AF standards subjected to the same TFA derivatization procedure. Extracts were injected in duplicate throughout this method validation study to ensure maximum instrumental precision and to monitor injection repeatability within the matrix background. However, a single-injection workflow can be seamlessly adopted for high-throughput routine screening to optimize total analytical run time, provided that standard bracketing sequences are maintained to monitor system drift. To compensate for potential matrix effects, quantification was performed using matrix-matched calibration curves.

### 4.4. Method Performance and Validation

The performance of the optimized method was validated by assessing recovery, precision (expressed as relative standard deviation, RSD%), and sensitivity (LOD and LOQ), following the Eurachem Guide [22]. These protocols ensure the statistical robustness required to verify compliance with the performance criteria established by Commission Regulation (EU) 2023/2782 [23].

#### 4.4.1. Calibration and Matrix Effect

Calibration curves were constructed using a multi-component standard in acetonitrile at seven concentration levels ranging from 0.2 to 20 µg/kg (matrix equivalent). To evaluate the matrix effect (ME%), matrix-matched calibration curves were generated by fortifying blank pea flour extracts at the same concentration levels. The ME% was calculated as the ratio between the slope of the matrix-matched calibration curves and the slope of the solvent-based calibration curve minus 1, expressed as a percentage. To ensure consistency, all standards, whether in solvent or in matrix, underwent the same TFA derivatization procedure. Each level was analyzed in triplicate.

#### 4.4.2. Recovery and Precision

Recovery was assessed to evaluate the extraction efficiency of the MAE protocol and to determine the impact of the optional SPE cleanup on analyte loss. The pea flour (PF1) was fortified with a mixed AFs standard at 2 and 10 µg/kg (*n* = 3). Recovery was calculated as the percentage ratio between the peak area of the fortified sample and the peak area of a matrix-matched standard at the same concentration.

Intra-day precision (repeatability) was assessed by analyzing six independent replicates of spiked pea flour on the same day. Inter-day precision was evaluated by analyzing 6 replicates over two consecutive days (*n* = 3 per day). Both parameters were determined at three fortification levels (0.4, 2, and 10 µg/kg), covering the regulatory range of interest.

#### 4.4.3. LOD and LOQ

The LOD and LOQ were estimated according to Eurachem guidelines [22] using the standard deviation of the lowest spiked level (0.2 µg/kg, *n* = 9). The LOD and LOQ were calculated as 3 and 10 times, respectively, the standard deviation of the replicated measurements divided by the slope of the matrix-matched calibration curves.

#### 4.4.4. Statistical Analysis

Experimental data were processed using Microsoft Excel (version 16.75). To evaluate the statistical significance of the differences between the LOQs obtained via the direct MAE and the MAE + SPE protocols, an uncertainty-based decision rule was applied to assess metrological equivalence. The standard uncertainty of the LOQ (u_LOQ_) was derived from the standard error of the regression and the slope of the matrix-matched calibration curves. The expanded uncertainty (U) for each protocol was then calculated using a coverage factor of k = 2, providing a 95% confidence level.

A difference between the two protocols was considered statistically significant only if the absolute difference between the LOQs (|Δ_LOQ_|) exceeded the combined expanded uncertainty of the difference (U_comb_), calculated according to the Eurachem Guide [22] as: $U_{comb}=\sqrt{U_{MAE}^{2}+U_{MAE+SPE}^{2}}$, where U_MAE_ and U_MAE+SPE_ represent the individual expanded uncertainties (k = 2, 95% confidence level) of the respective protocols. If |Δ_LOQ_| < U_comb_, the two analytical boundaries were considered metrologically equivalent. The detailed numerical parameters, including slope, linear regression standard errors, and the final critical difference values for this test, are provided in Table S1 of the Supplementary Material.

## 5. Conclusions

This study demonstrates that MAE coupled with direct TFA derivatization represents a rapid, sustainable and highly efficient workflow for determining AFs in pea flour via HPLC-FLD, completely bypassing the need for conventional cleanup steps. The optimized protocol yields high precision and recoveries that are fully compliant with current European performance criteria. Head-to-head metrological comparison revealed that while SPE marginally increases recoveries, it does not significantly mitigate matrix effects or improve method sensitivity, as confirmed by statistically equivalent LOQ values.

The combination of MAE with micro-volume TFA treatment appears to exert a partial in situ cleanup effect, ensuring excellent baseline resolution. By eliminating the multi-step SPE cartridge phase, this direct approach drastically simplifies the analytical architecture, minimizes toxic solvent consumption, and enhances sample throughput. Although moderate matrix-induced signal suppression occurs for AFB_1_ and AFG_1_, this phenomenon is systematically and successfully corrected using matrix-matched calibration curves, making the method ideal for green routine screening.

While the analysis of commercial samples (*n* = 7) indicated a low occurrence of AFs, the limited sample size reflects the current niche status of pea-flour. This highlights the necessity for larger-scale, multi-regional market surveys in future work to comprehensively map contamination risks in expanding plant-based protein supply chains.

Overall, this validated strategy offers a leaner, more sustainable, and economically viable alternative for routine food safety monitoring.

## Figures and Tables

**Table 1:** Regression parameters (R^2^, intercept, and slope) and matrix effect (ME%) for AFG1, AFB1, AFG2, and AFB2 in solvent, pea flour extract (MAE-only), and purified extract (MAE + SPE). Matrix effects were calculated by comparing the slopes of matrix-matched curves against solvent-based calibration.

Calibration Conditions	AF	R^2^	Intercept	Slope	ME%
Solvent (water/acetonitrile 90:10, *v*/*v*)	G1	0.9999	−0.017	0.389	-
	B1	0.9999	−0.047	1.102	-
	G2	1.0000	−0.007	0.648	-
	B2	0.9999	−0.063	1.372	-
Matrix-matched MAE-only	G1	0.9993	0.026	0.293	−25
	B1	0.9999	−0.004	0.846	−23
	G2	0.9999	0.063	0.580	−11
	B2	1.0000	0.044	1.220	−11
Matrix-matched MAE + SPE	G1	0.9998	−0.005	0.277	−29
	B1	0.9998	−0.067	0.866	−21
	G2	0.9985	0.154	0.650	+0.4
	B2	0.9990	0.290	1.343	−2.1

**Table 2:** Intra- (RSD_r_%) and inter-day (RSD_R_%) repeatability expressed as relative standard deviation (*n* = 6) for aflatoxins in pea flour at three fortification levels (0.4, 2, and 10 µg/kg) using MAE-only and MAE + SPE procedures. Recoveries are expressed as percentage and standard deviation of three replicates (REC% ± SD, *n* = 3) at two fortification levels (2 and 10 µg/kg).

Protocol	Level (µg/kg)	Parameter	Aflatoxin			
			G1	B1	G2	B2
MAE	0.4	RSD_r_%	5.1	4.2	4.6	3.1
		RSD_R_%	6.8	4.1	4.7	2.8
	2.0	RSD_r_%	2.1	3.1	2.9	1.1
		RSD_R_%	8.7	7.0	2.2	1.5
		REC% ± SD	89.7 ± 14.4	68.3 ± 15.3	74.6 ± 8.1	65.7 ± 12.6
	10.0	RSD_r_%	1.7	1.5	0.2	0.9
		RSD_R_%	6.4	5.6	0.4	1.2
		REC% ± SD	70.2 ± 8.0	72.2 ± 7.8	73.0 ± 0.8	71.9 ± 1.1
MAE + SPE	0.4	RSD_r_%	2.1	6.6	3.4	1.0
		RSD_R_%	2.4	6.0	2.8	0.8
	2.0	RSD_r_%	3.6	0.7	2.7	0.6
		RSD_R_%	3.2	1.9	2.1	1.9
		REC% ± SD	95.2 ± 12.8	98.8 ± 1.9	83.5 ± 4.1	83.1 ± 2.7
	10.0	RSD_r_%	1.6	0.5	1.2	0.4
		RSD_R_%	2.7	2.8	0.9	1.5
		REC% ± SD	103 ± 11	98.9 ± 7.0	95.2 ± 4.6	98.8 ± 5.0

**Table 3:** Limits of detection (LOD) and quantification (LOQ) obtained by using the two protocols (MAE and MAE + SPE), along with the absolute difference in LOQ (|Δ_LOQ_|) and the combined uncertainty (U_comb_).

Aflatoxin	LOD (µg/kg)		LOQ (µg/kg)		|Δ_LOQ_|	U_comb_
	MAE	MAE + SPE	MAE	MAE + SPE		
G1	0.07	0.13	0.25	0.43	0.19	0.45
B1	0.05	0.07	0.15	0.23	0.08	0.23
G2	0.04	0.05	0.12	0.18	0.05	0.58
B2	0.03	0.04	0.09	0.12	0.03	0.44

**Table 4:** Sample coding, place of production, market placement, and compositional characteristics.

Sample Code	Place of Production	Market Placement	Proteins (g/100 g)	Fibers (g/100 g)	Fats (g/100 g)	Carbohydrates (g/100 g)
FP1	Italy	Italian	22.0	14.0	1.5	48.0
FP2	Italy	Italian	24.6	14.7	1.2	45.6
FP3	Italy	Italian	23.6	9.4	2.1	62.2
FP4	Italy	Italian	19.0	6.3	2.0	60.0
FP5	Italy	Italian	21.0	18.0	0.9	44.0
FP6	Italy	Italian	20.0	20.0	2.1	45.0
FP7	Slovenia	Slovenian	24.0	8.1	2.0	52.0
FP8	Outside of the EU	Slovenian	n.r.	n.r.	n.r.	n.r.

n.r.: not reported (sample purchased in bulk, lacking nutritional labelling).

## Data Availability

The data presented in this study are available on request from the corresponding author.

## Supplementary Materials

The following supporting information can be downloaded at: https://www.mdpi.com/article/10.3390/molecules31122035/s1, Figure S1: Overlaid HPLC-FLD chromatographic traces obtained from the analysis of a fortified pea flour extract (2 µg/kg for each analyte), comparing a standard solution in solvent (green line), a direct microwave-assisted extract (red line, “MAE-only”), and an SPE-purified extract (blue line, “MAE + SPE”); Figure S2: Representative HPLC-FLD chromatogram of the unfortified pea flour matrix blank (“MAE-only” protocol), displayed at an optimized signal attenuation to demonstrates the total absence of endogenous aflatoxins or interfering matrix co-solutes within the specific retention time windows of the target analytes. Table S1: Detailed metrological parameters, individual expanded uncertainties (U), and combined expanded uncertainties (Ucomb) used for the critical difference test of LOQ.
