# Supplementary material for: Evaluation of the Necessity of a Cleanup Step After Microwave-Assisted Extraction (MAE) of Aflatoxins in Pea Flour
Source: Molecules. 2026 Jun 10;31(12):2035. doi: 10.3390/molecules31122035 (PMC13304597; doi:10.3390/molecules31122035)
Supplement: Supplementary file 1 [file molecules-31-02035-s001.zip › molecules-4252631-supplementary.pdf]

*Supplementary Material*

# Evaluation of the Necessity of a Cleanup Step After Microwave-Assisted Extraction (MAE) of Aflatoxins in Pea Flour

**Laura Barp *, Elisa Denittis, Chiara Conchione and Sabrina Moret**

Department of Agri-Food, Environmental and Animal Science, University of Udine, via Sondrio 2/a, 33100 Udine, Italy; denittis.elisa@spes.uniud.it (E.D.); chiara.conchione@uniud.it (C.C.); sabrina.moret@uniud.it (S.M.)
* Correspondence: laura.barp@uniud.it

**Table S1.** Detailed metrological parameters, individual expanded uncertainties (U), and combined expanded uncertainties ($U_{comb}$) used for the critical difference test of LOQ. Specifically, the calibration curve slope was obtained via linear regression of the matrix-matched MAE-only and MAE+SPE calibration data. The standard error of the regression (residual standard deviation), representing the overall vertical dispersion of the experimental data points around the calculated linear regression line. The standard uncertainty of the quantification limit ($u_{LOQ}$) was calculated directly as the ratio between the residual standard error and the slope. The individual expanded measurement uncertainty (U (k=2)) for each independent protocol was calculated by multiplying the standard uncertainty ($u_{LOQ}$) by a coverage factor k = 2, corresponding to a 95% confidence level. The absolute experimental difference between the limits of quantification obtained with the two distinct protocols was calculated as $|\Delta_{LOQ}| = |LOQ_{MAE} - LOQ_{MAE+SPE}|$
The combined expanded uncertainty of the difference between two independent analytical results ($U_{comb}$) was calculated by pooling the individual expanded uncertainties according to the Eurachem Guide guidelines and the error propagation law ($U_{comb} = \sqrt{U_{MAE}^2 + U_{MAE+SPE}^2}$).

| Protocol | Aflatoxin | Slope | Residual standard error | $u_{LOQ}$ | U (k=2) | $\lvert\Delta_{LOQ}\rvert$ | $U_{comb}$ |
|---|---|---|---|---|---|---|---|
| MAE-only | G1 | 0.29 | 0.04 | 0.12 | 0.24 | 0.19 | 0.45 |
| | B1 | 0.85 | 0.06 | 0.07 | 0.14 | 0.08 | 0.23 |
| | G2 | 0.58 | 0.05 | 0.08 | 0.16 | 0.05 | 0.58 |
| | B2 | 1.22 | 0.03 | 0.03 | 0.05 | 0.03 | 0.44 |
| MAE+SPE | G1 | 0.27 | 0.05 | 0.19 | 0.38 | | |
| | B1 | 0.87 | 0.08 | 0.09 | 0.19 | | |
| | G2 | 0.65 | 0.18 | 0.28 | 0.55 | | |
| | B2 | 1.34 | 0.30 | 0.22 | 0.44 | | |

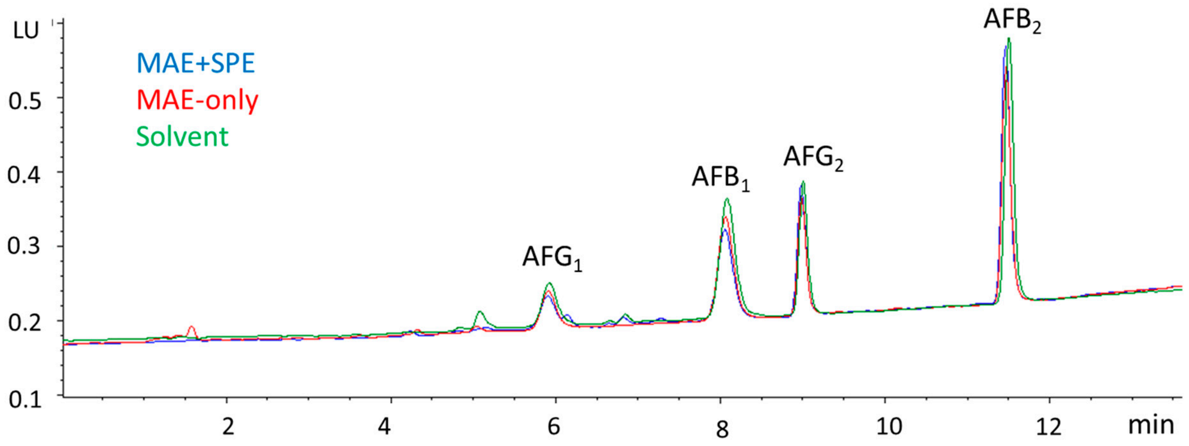


**Figure S1.** Overlaid HPLC-FLD chromatographic traces obtained from the analysis of a fortified pea flour extract (2 µg/kg for each analyte), comparing a standard solution in solvent (green line), a direct microwave-assisted extract (red line, "MAE-only"), and an SPE-purified extract (blue line, "MAE+SPE").

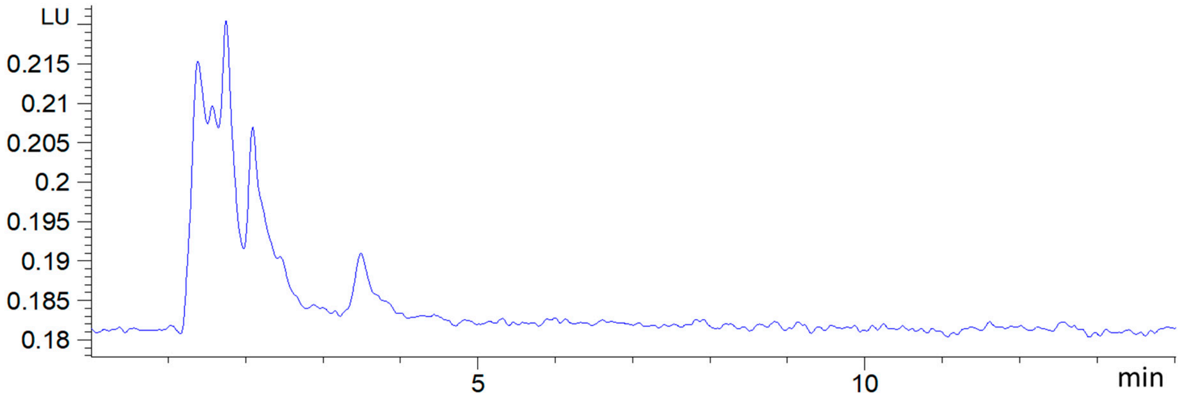


**Figure S2.** Representative HPLC-FLD chromatogram of the unfortified pea flour matrix blank ("MAE-only" protocol), displayed at an optimized signal attenuation to demonstrates the total absence of endogenous aflatoxins or interfering matrix co-solutes within the specific retention time windows of the target analytes.
